# The Predictive Role of Executive Functions and Psychological Factors on Chronic Pain after Orthopaedic Surgery: A Longitudinal Cohort Study

**DOI:** 10.3390/brainsci10100685

**Published:** 2020-09-28

**Authors:** Emanuele Maria Giusti, Chiara Manna, Giorgia Varallo, Roberto Cattivelli, Gian Mauro Manzoni, Samantha Gabrielli, Federico D’Amario, Marco Lacerenza, Gianluca Castelnuovo

**Affiliations:** 1 Department of Psychology, Catholic University of Milan, 20123 Milan, Italy; chiaramanna1993@gmail.com (C.M.); giorgia.varallo@unicatt.it (G.V.); roberto.cattivelli@unicatt.it (R.C.); gianluca.castelnuovo@unicatt.it (G.C.); 2 Istituto Auxologico Italiano IRCCS, Psychology Research Laboratory, Ospedale San Giuseppe, 28921 Verbania, Italy; 3 Faculty of Psychology, eCampus University, 22060 Novedrate, Italy; gianmauro.manzoni@uniecampus.it; 4 Casa di Cura San Pio X, 20159 Milan, Italy; samanthagabrielli@outlook.com; 5 Humanitas San Pio X Clinic, 20159 Milan, Italy; federico.damario@sanpiox.humanitas.it; 6 Neurology Service, and Pain Center, Humanitas San Pio X Clinic, 20159 Milan, Italy; lacerenzam@gmail.com

**Keywords:** chronic post-surgical pain, psychology, catastrophizing, depression, anxiety, predictors, executive functions, rehabilitation

## Abstract

Prevention and treatment of chronic post-surgical pain should be based on the early identification of patients at risk. The presence of a deficit in executive functions, along with the presence of psychological risk factors, could impair the use of appropriate pain coping strategies and might facilitate the transition to chronic post-surgical pain. A longitudinal cohort study was implemented. Patients listed for orthopaedic surgery were enrolled. Variables measured before surgery were pain intensity, the sensory, affective, cognitive and mixed components of pain, state and trait variables associated with the psychological status of the patient, fear of movement, pain catastrophizing, visual attention and cognitive flexibility. Pain intensity and the components of pain were re-evaluated after surgery and after three months. A linear mixed model was used to assess the predictors of pain intensity, and a multivariate linear mixed model was used to assess the predictors of the pain components. 167 patients were enrolled. Controlling for sex, age, pain duration and surgical procedure, catastrophizing and visual attention were predictors of pain intensity at follow-up. The sensory component of pain was predicted by state anxiety, healthcare-related fears, pain catastrophizing and visual attention. Anxiety and catastrophizing were predictors of the affective and evaluative components of pain. The mixed component of pain was predicted by state anxiety, healthcare-related fears and pain catastrophizing. Executive functions, along with psychological risk factors, shape the course of post-surgical pain. The efficacy of preventive and rehabilitation treatment could be possibly enhanced if these factors are treated.

## 1. Introduction

Post-surgical pain arising after orthopaedic procedures has a high incidence, which can reach nearly half of the patients after three and six months from surgery [1,2]. This constitutes a major issue for patients, for health professionals and for the society, as it might lead to long-lasting disability, reduced quality of life and healthcare-related costs [3].

The role of psychological predictors on the transition to chronic pain after orthopaedic surgery has been proposed and found extensive corroboration [4,5,6]. Factors such as pain catastrophizing, depression, anxiety and fear of movement are known to be related to pain through neurobiological, psychological and behavioural mechanisms [7]. However, most of the studies focus on trait psychological characteristics, often overlooking the role of variables related to the presurgical transitory status of the patient. According to a recent systematic review with meta-analysis, these factors are rarely studied but might have a major role in the development of chronic pain after surgery, since it is linked to a specific event [5].

It has also been hypothesized that executive functions, i.e., a set of interrelated cognitive abilities encompassing inhibitory ability, working memory, and cognitive flexibility [8], might also play a role in the development of chronic post-surgical pain. In particular, impairment in executive functions could weaken the ability to flexibly select effective coping strategies to manage pain and associated psychological states. Correlational studies suggest that, in laboratory settings, painful stimulation reduces executive functioning performance [9]. Regarding post-surgical pain, a longitudinal cohort study by Attal et al. assessed the role of cognitive flexibility, visual memory and attention on 89 patients undergoing total knee arthroplasty and 100 patients undergoing breast surgery, finding that cognitive flexibility and visual memory were linked to both pain intensity and the presence of neuropathic pain [10]. Additional evidence is however needed to confirm the role of executive functions as predictors of chronic post-surgical pain.

It is to be noted that chronic pain is a complex and multidimensional condition, which cannot be reduced only to its intensity. Its sensory, affective and evaluative components all contribute to the overall experience of the sufferer and, since each of them is linked to different neural pathways, it can be hypothesized that psychological factors and executive functions might have a differential role on each of them [11,12]. Knowledge about the predictors of these components might help to develop more targeted rehabilitation treatments [13].

Therefore, we aimed to evaluate the role of state and trait psychological variables and executive functions on pain after orthopaedic surgery, taking into account both its intensity and its components.

## 2. Materials and Methods

A longitudinal cohort study was performed. Patients listed for orthopaedic surgery at the hospital “San Pio X”, Milan, Italy, were considered for enrolment. The inclusion criteria were age ≤80 years and being listed for orthopaedic procedures at the Institution. Exclusion criteria were physical or mental inability to provide signed informed consent. Enrolment was initiated in October 2014 and was concluded in December 2015.

### 2.1. Procedure

Patients were enrolled after they had attended the pre-surgical visits. Median time from the pre-surgical visits and surgery was 7 days (range 4–25). Patients who agreed to participate were asked to complete a battery of questionnaires evaluating pain intensity, pain components and their potential predictors and to undertake a brief neuropsychological assessment. On the third day after surgery, patients were reached in their room and were asked to fill the post-surgical battery of questionnaires which included the scales measuring pain intensity and pain components. Finally, patients were contacted after three months from surgery with a phone call and the scales measuring pain intensity and pain components were re-administered to assess chronic post-surgical pain [14]. All patients filled an informed consent form. The procedures of this research were approved by the Institutional Ethics Committee (code 52617). All procedures in the present study were in accordance with the ethical standards of the Helsinki Declaration of 1969 and its later amendments.

### 2.2. Measurement Instruments

The following measurement instruments were used at baseline:

- A Numeric Rating Scale (NRS) measuring pain intensity ranging from 0 (“No pain”) to 10 (“Worst possible pain”) [15].
- The Italian Pain Questionnaire (IPQ) [16]. The IPQ assesses the sensory, affective and evaluative components of the patient’s pain experience. It is the Italian version of the McGill Pain Questionnaire, which was translated and modified to overcome the cross-cultural differences of the semantics of pain. The IPQ includes 42 pain descriptors divided into four main classes (sensory, affective, evaluative and mixed), and 16 sub-classes corresponding to those of the McGill Pain Questionnaire. Every sub-class has a variable number (from 2 to 5) of descriptors in ascendant order and every descriptor has a corresponding value in the scoring of the respective component based on its position. The average of the scoring of the descriptors of each component is named Pain Rating Index (PRI). Therefore, four PRI were calculated, namely PRI-Sensory (PRI-S), PRI-Affective (PRI-A), PRI-Evaluative (PRI-E) and PRI-Mixed (PRI-M).
- The Pain Catastrophizing Scale (PCS) [17]. The PCS assesses the frequency of thoughts and feelings associated with pain using 13 items on a 5-point Likert scale (from 0 = “not at all” to 4 = “all the time”). The Italian version of PCS has shown good reliability and construct validity [18]. In this study, its total score was employed [19]. Higher scores in the PCS indicate higher levels of pain catastrophizing.
- The Tampa Scale of Kinesiophobia (TSK) [20,21]. The TSK assesses pain-related fear of movement and re-injury using 17 items on a 4-point Likert scale (from 1 = “completely disagree” to 4 = “completely agree”). The TSK includes two subscales: Harm (TSK-H), which assesses beliefs that there is something wrong with the body, and Avoidance of activities (TSK-A), which assesses beliefs that avoiding exercise or activities might prevent an increase in pain. Higher values indicate a higher fear of movement. The Italian version showed good psychometric properties [20].
- Card A and B of the Cognitive-Behavioral Assessment—Hospital form (CBA-H) [22]. The CBA-H was developed to evaluate the psychological status of patients with physical illnesses, taking into account both state and trait variables. Card A includes 21 items focusing on the psychological status at the moment of administration and includes three subscales, namely State Anxiety (CBA-H-A), Healthcare-related Fears (CBA-H-HF) and Situational Depressive reactions (CBA-H-SD). Card B includes 23 items focusing on the three months preceding the moment of administration and examines Depressive mood (CBA-H-D), Psychophysical Stress (CBA-H-PS) and psychophysical Well-Being (CBA-H-WB). The responses are coded using a dichotomous yes/no response. The scale showed adequate reliability and good structural and construct validity [23].
- The Trail Making Test (TMT) [24]. The TMT is a neuropsychological test which includes two parts. In part A (TMT-A), the patient is required to connect numbered circles drawing consecutive lines. In part B (TMT-B), the patient is required to connect consecutively numbered letters and circles, alternating between letters and numbers. Time to complete the TMT-A is considered a measure of attention and visual scanning. Time to complete the TMT-B is considered a measure of set-shifting and cognitive flexibility. Italian norms were employed to adjust the raw scores taking into account age and education [25].

The measurement instruments employed after surgery and at follow-up were the NRS and the IPQ.

### 2.3. Statistical Analyses

Categorical data are described using counts and percentages, continuous data are described using means and standard deviations or, in case of non-normality, medians and interquartile ranges. Differences in demographic and clinical variables between surgical procedures were ascertained using chi-square tests, Mann–Whitney tests or t-tests, as appropriate.

Amount, patterns and mechanisms of missing data were analysed using counts, statistical tests (chi-square, Mann–Whitney tests or t-tests to assess predictors of non-response) and graphical tools. Since attrition at follow-up was high, a multiple imputation procedure with predictive mean matching (20 datasets, 50 iterations) was conducted. This approach was chosen since multiple imputations, contrarily to listwise deletion, reduces bias in the estimated regression coefficients while maintaining the original relationships among variables [26]. Quality of imputed values was ascertained using graphical tools. Imputed datasets were used in the following analyses.

Predictors of pain intensity were assessed using a linear mixed model, whereas predictors of the components of pain were assessed using a multivariate mixed model. In these models, the repeated measures, i.e., at baseline, after surgery and at follow-up, of pain intensity and pain components were included as outcomes. Predictors were the variables related to the psychological status of the patient, pain catastrophizing, fear of movement, attention and cognitive flexibility. To control for confounding, fixed effects of sex, age, pain duration (less than one month, less than one year and more than one year, transformed into two dummy variables with less than one month as the reference group) and surgical area (knee, foot, shoulder and hip, transformed into three dummy variables with the knee as the reference group) were also added. In all the models, a random intercept and a random slope for the time were specified, indicating that the level and development of pain and pain components over time was modelled for each subject. The effect of predictors on follow-up pain was investigated by the inclusion of an interaction between the follow-up assessment and standardized predictors. Results are interpreted as per SD increase in the predictors and the association with change in pain from baseline to follow-up while taking into account the effect of the other variables in the model.

The significance threshold was set at *p* < 0.05. The multiple imputation procedure was performed using R (version 4.0.2) package mice [27], whereas mixed models were performed using the package nlme [28].

## 3. Results

### 3.1. Participants Characteristics

One hundred sixty-seven patients were enrolled. The demographic and clinical characteristics of the participants are reported in Table 1. Seventy-six patients underwent knee surgery (40 unicompartmental knee arthroplasty, 29 total knee arthroplasty and 7 meniscectomy), 60 patients underwent foot surgery (hallux valgus surgery), 20 patients underwent shoulder arthroplasty and 11 patients underwent hip replacement surgery.

One hundred forty-two patients filled the post-surgical battery and 104 filled the follow-up battery. 46 patients (44%) had pain intensity ≥3 at follow-up. The main reasons for dropout after surgery were rescheduling of surgery, early discharge and withdrawal of consent. The main reasons for dropout at follow-up were unavailability at phone calls and withdrawal of consent. Missing data at baseline was negligible. Age was associated with missing data in the questionnaires administered after surgery and at follow-up. Therefore, data were deemed to follow a Missing At Random mechanism. A multiple imputation procedure was employed to account for the missing data regarding pain intensity and pain characteristics after surgery and at follow-up. The multiple imputation procedure converged, and the distributions of imputed data were consistent with those of the measured data. The imputed datasets were employed in the following analyses.

### 3.2. Predictors of Pain Intensity

After adjusting for sex, age, surgical area, pain duration and for the baseline values of pain intensity, pain intensity at follow-up was predicted only by pain catastrophizing and by visual attention as measured by the TMT-A. Coefficients of the interactions between time and each predictor are reported in Table 2.

### 3.3. Predictors of the Components of Pain

Results of the multivariate linear mixed model showed that, after controlling for age, sex, surgical area, pain duration and the baseline measurements of pain components, the sensory component of follow-up pain was predicted by state anxiety, healthcare-related fears, pain catastrophizing and visual attention; the affective and evaluative components of pain were predicted by state anxiety and pain catastrophizing; the mixed components of pain were predicted by state anxiety, healthcare-related fears and pain catastrophizing. Standardized coefficients of the interaction between time and predictors are reported in Table 3.

## 4. Discussion

This study aimed to assess the predictive role of executive functions and psychological factors on pain intensity and pain characteristics after orthopaedic surgery. The results show that pain intensity was predicted by visual attention and pain catastrophizing, that the sensory component of pain was predicted by pain catastrophizing, state anxiety, healthcare-related fears and visual attention; that state anxiety and pain catastrophizing were predictors of the affective and evaluative components of pain, and that the mixed component of pain was predicted by state anxiety, healthcare-related fears and pain catastrophizing.

Trait psychological characteristics, such as pain catastrophizing, are known to be significant predictors of post-surgical pain [5,29,30], and this was corroborated by the present study. Conversely, the predictive role of state variables has been rarely addressed. In the present study, pre-surgical state anxiety was not associated with post-surgical pain intensity but was a significant predictor of the sensory, affective, evaluative and mixed components of pain at follow-up. Healthcare-related fears were also associated with the sensory and mixed component of pain. The predictive effect of state anxiety has been noted elsewhere [31,32,33]. Depressive reactions, conversely, did not affect the incidence of post-surgical pain. Taken together, these findings suggest that the presurgical state of fear and anxiety of the patient regarding the potential complications of surgery might trigger exaggerated responses during the postsurgical period, leading to less effective coping strategies.

In this study, it was found that visual attention was related to pain intensity at follow-up. It is known that chronic pain has an influence on the efficiency of cognitive functions and, in particular, of executive functions, and that this could trigger a vicious circle [34]. It was hypothesized that the presence of pre-surgical impairment of executive functions could also trigger the transition to chronic post-surgical pain, but this hypothesis has been rarely assessed [5,10]. The presence of deficits in attention abilities, in particular, could make it more difficult for the patient to engage in complex tasks and might increase the tendency to be hypervigilant as a response to pain experiences [35].

These findings have several clinical implications. Firstly, a pre-surgical psychological assessment of patients is warranted to identify the ones at risk for an unfavourable outcome. Brief self-report questionnaires are available for the assessment of factors such as pain catastrophizing and state anxiety, which could be measured during the pre-surgical visit and might allow to plan timely treatment of the patients in case of complications. Psychological therapies are known to be effective for the treatment of pain [36] and to reduce the associated costs [37]. Brief psychological treatments developed to treat the patient during the perioperative period are already available and should be targeted based on the characteristic of the patient [38].

The main limitation of this study is the heterogeneity of the sample since patients undergoing different surgical procedures were enrolled. In addition, the presence of past or comorbid conditions, such as brain injury, and medication intake were not assessed and controlled for. Finally, attrition at follow-up was high and this could have increased the possibility of the presence of selection bias. Statistical procedures, such as controlling for the surgical are and the use of multiple imputations could only partly overcome these limitations.

## 5. Conclusions

Pain catastrophizing is an important predictor of post-surgical pain intensity and its components and, along with pre-surgical state anxiety and healthcare-related fears, should be taken into account to identify patients at risk for chronic post-surgical pain and to provide targeted psychological treatment. Visual attention was found as a predictor of pain intensity and the sensory component of pain, and studies are needed to deepen our knowledge about the mechanisms that link executive functions with the patients’ pain experience.

## Figures and Tables

**Table 1:** Demographic and clinical characteristics of the participants of the study.

			Surgical Area				
Knee (*n* = 76)		Total (*n* = 167)		Foot (*n* = 60)	Shoulder (*n* = 20)	Hip (*n* = 11)	*p* *
Sex	Male	47 (28.1)	26 (34.2)	6 (10)	11 (55)	4 (36.4)	
	Female	120 (71.9)	50 (65.8)	54 (90)	9 (45)	7 (63.6)	<0.01
Age		55 (15.5)	54.2 (18.2)	53.8 (12.6)	55.6 (13.6)	66.3 (8.7)	0.09
Occupation	Unemployed	26 (15.6)	12 (15.8)	10 (16.7)	1 (5.0)	3 (27.3)	
	Worker	90 (53.9)	41 (53.9)	35 (58.3)	12 (60.0)	2 (18.2)	
	Retired	51 (30.5)	23 (30.3)	15 (25.0)	7 (35.0)	6 (54.5)	0.22
Civil status	Single	32 (19.2)	20 (26.3)	8 (13.3)	4 (20.0)	0 (0.0)	
	Coupled or married	106 (63.5)	40 (52.6)	44 (73.3)	15 (75.0)	7 (63.6)	
	Divorced	6 (3.6)	3 (3.9)	2 (3.3)	1 (5.0)	0 (0.0)	
	Widow	23 (13.8)	13 (17.1)	6 (10.0)	0 (0.0)	4 (36.4)	0.06
Education	Elementary or middle school	53 (31.7)	24 (31.6)	12 (20.0)	10 (50.0)	7 (63.6)	
	High school	80 (47.9)	38 (50.0)	31 (51.7)	7 (35.0)	4 (36.4)	
	University	34 (20.4)	14 (18.4)	17 (28.3)	3 (15.0)	0 (0.0)	0.03
Pain duration	Less than one month	12 (7.2)	9 (11.8)	3 (5.0)	0 (0.0)	0 (0.0)	
	Less than one year	57 (34.1)	33 (43.4)	9 (15.0)	12 (60.0)	3 (27.3)	
	More than one year	98 (58.7)	34 (44.7)	48 (80.0)	8 (40.0)	8 (72.7)	<0.01
CBA-H-A		2.5 (2.7)	2.7 (2.8)	2.1 (2.5)	2 (2.4)	3.9 (3.3)	0.14
CBA-H-HF		1.3 (1.2)	1.4 (1.3)	1.3 (1.2)	1.1 (1.2)	1.5 (1.1)	0.8
CBA-H-SD		0.5 (0.9)	0.6 (0.9)	0.4 (0.8)	0.6 (1)	0.7 (0.8)	0.64
CBA-H-D		3.2 (2.7)	3.5 (2.8)	2.4 (2.4)	2.8 (2.4)	5.5 (3)	<0.01
CBA-H-WB		3.5 (1.9)	3.4 (1.9)	3.9 (1.8)	3.1 (2)	2.4 (1.9)	0.07
CBA-H-PS		2.7 (2)	2.7 (1.9)	2.5 (2.2)	2 (2)	3.8 (2.2)	0.13
PCS		16 (10)	15.9 (9.9)	16.9 (10.2)	17.6 (10.1)	21.7 (8.8)	0.31
TSK-H		2.3 (0.7)	2.4 (0.5)	2 (0.7)	2.4 (0.8)	2.7 (0.6)	<0.01
TSK-A		2.2 (0.8)	2.4 (0.8)	1.8 (0.7)	2.5 (0.7)	3 (0.7)	<0.01
TMT-A		39 (23.2)	39.7 (24)	35.7 (17)	34.6 (18.1)	59.6 (41.6)	0.01
TMT-B		90.7 (44.4)	94 (49.1)	88.9 (32.1)	83 (39)	91.5 (73.7)	0.78
NRS		6 (2.4)	5.6 (2.5)	6.2 (2.4)	6 (2.5)	6.8 (1.8)	0.31
PRI-S		0.2 [0.1, 0.3]	0.2 [0.1, 0.3]	0.2 [0.1, 0.3]	0.2 [0.1, 0.3]	0.3 [0.2, 0.3]	0.51
PRI-A		0.1 [0.0, 0.3]	0.1 [0.0, 0.2]	0.1 [0.0, 0.2]	0.1 [0.0, 0.3]	0.3 [0.3, 0.4]	0.01
PRI-E		0.1 [0.0, 0.3]	0.1 [0.1, 0.2]	0.1 [0.0, 0.3]	0.1 [0.0, 0.3]	0.3 [0.1, 0.4]	0.23
PRI-M		0.1 [0.0, 0.2]	0.1 [0.0, 0.2]	0.1 [0.0, 0.2]	0.2 [0.0, 0.3]	0.2 [0.0, 0.3]	0.37
**Variables assessed after surgery (n = 142)**							
NRS		4.7 (3.1)	4.8 (2.9)	4.7 (3.3)	4.7 (3)	3.7 (3.1)	0.79
PRI-S		0.2 [0.1, 0.4]	0.2 [0.1, 0.3]	0.2 [0.1, 0.4]	0.2 [0.1, 0.4]	0.1 [0.1, 0.2]	0.67
PRI-A		0.1 [0.0, 0.3]	0.1 [0.0, 0.3]	0.1 [0.0, 0.2]	0.1 [0.0, 0.3]	0.1 [0.0, 0.3]	0.97
PRI-E		0.1 [0.0, 0.3]	0.1 [0.1, 0.3]	0.1 [0.0, 0.3]	0.1 [0.0, 0.2]	0 [0.0, 0.1]	0.51
PRI-M		0 [0.0, 0.2]	0 [0.0, 0.2]	0 [0.0, 0.2]	0 [0.0, 0.2]	0 [0.0, 0.2]	0.70
**Variables assessed at follow-up (n = 104)**							
NRS		2.5 (2.7)	2.7 (2.8)	1.7 (2.6)	3.5 (2.5)	2.2 (1.5)	0.21
PRI-S		0.1 [0.0, 0.2]	0.1 [0.1, 0.2]	0 [0.0, 0.1]	0.1 [0.1, 0.2]	0.1 [0.1, 0.1]	0.02
PRI-A		0 [0.0, 0.1]	0 [0.0, 0.2]	0 [0, 0]	0 [0.0, 0.2]	0 [0, 0]	0.44
PRI-E		0.1 [0.0, 0.7]	0.2 [0.0, 2.3]	0 [0.0, 0.2]	0.4 [0.1, 1.0]	1.2 [0.7, 2.2]	0.01
PRI-M		0 [0.0, 0.9]	0 [0.0, 1.2]	0 [0.0, 0.1]	0 [0.0, 1.2]	0.9 [0.0, 1.1]	0.25

Note. Descriptive statistics are frequencies and percentages for categorical variables, medians [IQR] for continuous non-normal variables and means (sd) for continuous normal variables. * *p* values are calculated using chi-square tests in case of categorical variables, Mann–Whitney tests in case of continuous non-normal continuous variables, and **t**-tests in case of continuous normal variables. Abbreviations: CBA-H-A: Cognitive-Behavioral Assessment–Anxiety subscale; CBA-H-HF: Cognitive-Behavioral Assessment–Health-related Fears subscale; CBA-H-SD: Cognitive-Behavioral Assessment —Situational Depressive reactions subscale; CBA-H-D: Cognitive-Behavioral Assessment–Depressive mood subscale; CBA-H-WB: Cognitive-Behavioral Assessment–psychophysical Well-Being subscale; CBA-H-PS: Cognitive-Behavioral Assessment–Psychophysical Stress subscale; PCS: Pain Catastrophizing Scale; TSK-H: Tampa Scale of Kinesiophobia–Harm subscale; TSK-A: Tampa Scale of Kinesiophonia–Avoidance of activities subscale; TMT-A: Trail Making Test–part A; TMT-B: Trail Making Test–part B; NRS: Numeric Rating Scale; PRI-S: Pain Rating Index–Sensory component; PRI-A: Pain Rating Index–Affective component; PRI-E: Pain Rating Index–Evaluative component; PRI-M: Pain Rating Index–Mixed component.

**Table 2:** Results of the linear mixed model performed to assess the predictors of pain intensity.

	B	Se	*p*
Sex	0.056	0.129	0.67
Age	−0.003	0.004	0.49
Area—Feet ^a^	0.139	0.119	0.24
Area—Shoulder ^a^	−0.124	0.167	0.46
Area—Hip ^a^	−0.624	0.209	<0.01 *
Pain duration—less than one year ^b^	0.225	0.277	0.42
Pain duration—more than one year ^b^	0.231	0.271	0.40
CBA-H-A	−0.108	0.086	0.21
CBA-H-HF	0.020	0.081	0.80
CBA-H-SD	−0.070	0.059	0.24
CBA-H-D	0.080	0.083	0.33
CBA-H-WB	0.002	0.062	0.97
CBA-H-PS	−0.022	0.065	0.74
PCS	0.401	0.070	0.00 *
TSK-H	−0.007	0.060	0.91
TSK-A	0.102	0.069	0.14
TMT-A	0.169	0.074	0.03 *
TMT-B	−0.050	0.070	0.47

Note. Abbreviations: CBA-H-A: Cognitive-Behavioral Assessment—Anxiety subscale; CBA-H-HF: Cognitive-Behavioral Assessment–Health-related Fears subscale; CBA-H-SD: Cognitive-Behavioral Assessment–Situational Depressive reactions subscale; CBA-H-D: Cognitive-Behavioral Assessment–Depressive mood subscale; CBA-H-WB: Cognitive-Behavioral Assessment–psychophysical Well-Being subscale; CBA-H-PS: Cognitive-Behavioral Assessment–Psychophysical Stress subscale; PCS: Pain Catastrophizing Scale; TSK-H: Tampa Scale of Kinesiophobia—Harm subscale; TSK-A: Tampa Scale of Kinesiophonia—Avoidance of activities subscale; TMT-A: Trail Making Test–part A; TMT-B: Trail Making Test–part B. ^a^ Reference group: patients who underwent knee surgery. ^b^ Reference group: patients who pain for less than one month. * *p* values are calculated using chi-square tests in case of categorical variables.

**Table 3:** Results of the multivariate mixed model performed to assess the predictors of pain components.

	PRI-S			PRI-A			PRI-E			PRI-M		
	B	se	*p*	B	se	*p*	B	se	*p*	B	se	*p*
Sex	0.019	0.094	0.84	0.021	0.101	0.83	0.002	0.095	0.98	−0.056	0.103	0.59
Age	−0.002	0.003	0.37	0.000	0.003	0.94	−0.003	0.003	0.36	0.000	0.003	0.88
Area—Feet ^a^	−0.203	0.095	0.03 *	−0.009	0.100	0.93	−0.015	0.094	0.87	0.047	0.107	0.66
Area—Shoulder ^a^	−0.745	0.122	<0.01*	−0.588	.126	<0.01 *	−0.635	0.123	<0.01 *	−0.392	0.133	<0.01 *
Area—Hip ^a^	−0.799	0.155	<0.01 *	−0.347	0.157	0.03*	−0.547	0.165	<0.01*	−0.681	0.186	<0.01 *
Pain duration—less than one year ^b^	0.077	0.200	0.70	−0.283	0.222	0.21	−0.198	0.160	0.22	−0.311	0.228	0.18
Pain duration—more than one year ^b^	−0.047	0.201	0.82	−0.378	0.217	0.09	−0.285	0.165	0.09	−0.441	0.249	0.08
CBA-H-A	−0.233	0.068	<.001 *	−0.253	0.065	<0.01*	−0.273	0.062	<0.01 *	−0.373	0.070	<0.01 *
CBA-H-HF	−0.195	0.066	<0.01 *	−0.118	0.066	0.08	−0.100	0.062	0.11	−0.151	0.071	0.04 *
CBA-H-SD	−0.006	0.043	0.90	-0.059	0.048	0.22	0.006	0.043	0.89	0.010	0.052	0.85
CBA-H-D	−0.106	0.061	0.09	0.018	0.062	0.77	0.050	0.059	0.40	0.035	0.066	0.59
CBA-H-WB	0.052	0.050	0.30	−0.088	0.052	0.09	−0.011	0.049	0.83	0.015	0.059	0.80
CBA-H-PS	0.096	0.053	0.07	−0.022	0.056	0.70	0.020	0.053	0.70	0.137	0.063	0.03 *
PCS	1.058	0.060	<0.01 *	0.911	0.057	<0.01*	0.874	0.054	<0.01 *	0.888	0.059	<0.01 *
TSK-H	0.025	0.048	0.60	0.083	0.047	0.08	0.065	0.046	0.16	−0.017	0.050	0.73
TSK-A	−0.007	0.049	0.89	−0.033	0.052	0.53	0.002	0.048	0.97	0.062	0.054	0.26
TMT-A	−0.192	0.055	<0.01 *	−0.076	0.056	0.18	−0.037	0.053	0.49	0.028	0.060	0.65
TMT-B	0.051	0.050	0.31	0.021	0.053	0.69	0.024	0.049	0.62	−0.062	0.053	0.24

Note. Only estimates of the interaction between predictors and time t2 (vs baseline) are reported. * significant at *p* < 0.05. Abbreviations: PRI-S: Pain Rating Index—Sensory component; PRI-A: Pain Rating Index–Affective component; PRI-E: Pain Rating Index—Evaluative component; PRI-M: Pain Rating Index–Mixed component; CBA-H-A: Cognitive-Behavioral Assessment–Anxiety subscale; CBA-H-HF: Cognitive-Behavioral Assessment–Health-related Fears subscale; CBA-H-SD: Cognitive-Behavioral Assessment–Situational Depressive reactions subscale; CBA-H-D: Cognitive-Behavioral Assessment—Depressive mood subscale; CBA-H-WB: Cognitive-Behavioral Assessment–psychophysical Well-Being subscale; CBA-H-PS: Cognitive-Behavioral Assessment–Psychophysical Stress subscale; PCS: Pain Catastrophizing Scale; TSK-H: Tampa Scale of Kinesiophobia–Harm subscale; TSK-A: Tampa Scale of Kinesiophonia–Avoidance of activities subscale; TMT-A: Trail Making Test–part A; TMT-B: Trail Making Test–part B. ^a^ Reference group: patients who underwent knee surgery. ^b^ Reference group: patients who pain for less than one month.
